# Myofascial pain syndrome and SARS-CoV-2: a case series

**DOI:** 10.2217/pmt-2021-0044

**Published:** 2021-10-04

**Authors:** Jaldhi Patel, Saba Javed

**Affiliations:** ^1^Department of Anesthesiology, University of Texas Health & Sciences Center, Houston, TX, USA; ^2^Department of Pain Management, University of Texas Health & Sciences Center, Houston, TX, USA

**Keywords:** coronavirus and pain, COVID-19 and myofascial pain, COVID-19 side effects, long-term COVID-19 complications, myofascial pain syndrome

## Abstract

SARS-CoV-2 is a novel virus that has caused a plethora of dysfunctions and changes in the human body. Our goal in this case study series was to demonstrate the relationship that coronavirus has had in newly diagnosing patients with myofascial pain syndrome (MFPS). Medical records were obtained from a pain clinic that demonstrated the effects of this virus on patients who developed MFPS between March 2020 and December 2020. Chart reviews were performed and demonstrated patients who had a history of chronic pain had subsequent episodes of worsening exacerbations of pain, more specifically trigger points, after being diagnosed with coronavirus. MFPS and SARS-CoV-2 are proposed to be correlated amongst chronic pain patients. Potential pathological mechanisms include coronavirus-induced hypoxic muscle dysfunctions as well as psychological stress triggering pain receptors, leading to myofascial pain syndrome.

## Lay abstract

COVID-19, the disease caused by the SARS-CoV-2 virus, may cause many problems and changes in the human body. In this case series, we propose a relationship between COVID-19 and myofascial pain syndrome (MFPS), a kind of chronic muscle pain affecting connective tissue in the muscles. Medical records of people who developed MFPS after a diagnosis of COVID-19 between March 2020 and December 2020 were obtained from a pain clinic. Chart reviews demonstrated that the three people with chronic pain included in this case series had episodes of worsening pain, more specifically in focal points, after being diagnosed with COVID-19. COVID-19 is suggested to have affected the development of MFPS in these chronic pain patients. Potential mechanisms of this relationship include different types of stress leading to MFPS.

**Practice points**

- Patients who were previously followed in chronic pain clinic were diagnosed with SARS-CoV-2 by PCR between March 2020 and December 2020.
- During follow-up visits after recovering from SARS-CoV-2, these patients were found to have changes in their pain distribution.
- The new pain was characterized as tenderness along specific muscle bands which caused reproduction of symptoms when pressed and muscle weakness or changes from baseline.
- After obtaining thorough histories, physical exams and comprehensive evaluations of prior labs, a diagnosis of myofascial pain syndrome (MFPS) was made in each patient.
- Once appropriate treatment of MFPS was started, each patient reported improvement in pain by at least 25%.
- Possible pathologic mechanisms for SARS-CoV-2 induced MFPS include muscle dysfunction caused by generalized hypoxic states, traumatic psychological stress and variations in physical activity during the infected period.
- In the future, there can be prospective studies assessing patients with a history of SARS-CoV-2 who may develop MFPS.

Myofascial pain syndrome (MFPS) is a chronic pain condition that involves sensitive taut areas in muscles, referred to as trigger points, that may cause pain in the muscle belly itself or at a distant location [1]. The predominant feature of MFPS is the myofascial trigger point, which is a small, localized area of muscle contraction that is extremely tender on palpation. Typically, the diagnosis is made clinically based on identification and palpation of this myofascial trigger point. Features of the myofascial trigger point include taut bands within the muscle, exquisite tenderness at a point on a taut band, and reproduction of the patient's pain (Box 1). This condition can be caused by multiple factors such as constant overload or repetitive strain, postural dysfunction, psychological stress, trauma or spinal pathology [2,3]. The pathophysiology consists of any initiating event that causes an increase in acetylcholine release that will enhance depolarization at postjunctional membrane of muscle fibers. This causes a muscle contraction due to the increased release of calcium. As the same inciting event recurs, there is a continuous contracture of sarcomeres which forms a trigger point. It is also believed that the repetitive stimulation induces a sense of hypoxia within the muscle, which causes sensitization of nociceptors [4,5]. As evident by the traumatic nature of the disease, severe acute respiratory syndrome coronavirus 2 (SARS-CoV-2) has been found to have intermediate and long-term effects on fatigue, respiratory function and carditis. The pain-related symptoms including myalgia and arthalgias account for 36% in a recent study [6]. Along with these symptoms, it also has been shown to cause lower limb weakness due to possible sclerosis or hypoxia [7,8]. Herein, we report three cases where patients diagnosed with SARS-CoV-2 developed myofascial pain syndrome.

Box 1. **Features of myofascial trigger points.**

1. Taut band within the muscle
2. Exquisite tenderness at a point on the taut band
3. Reproduction of the patient's pain
4. Local twitch response
5. Referred pain
6. Weakness
7. Restriction range of motion
8. Autonomic signs (skin warmth or erythema, tearing, piloerection)

## Case patient #1

Patient is a 68-year-old overweight female who initially presented in June 2020 for management of chronic pain. Past medical history was significant for bipolar disorder, chronic obstructive pulmonary disease, diabetes mellitus type 2, gastroesophageal reflux disease and chronic pain consisting of back, pelvic, rectal and vaginal pain. At the time of examination, she was initially taking gabapentin 300 mg bid and methadone 2.5 mg in the morning, 5 mg in the afternoon, and 2.5 mg in the evening for pain management. A 12-point review of systems was positive for generalized myalgias and back pain. Due to inadequate pain control, dose adjustment was made with an increase in methadone to 5 mg in the morning, 5 mg in the afternoon and 2.5 mg in the evening. Subsequently, she presented for a follow-up visit and medication refill in September 2020, at which time her pain was well controlled. Subsequently, patient was diagnosed with SARS-CoV-2 in October 2020 by a PCR test and was hospitalized for 10 days. Her hospital course was remarkable for high-dose oxygen therapy with CPAP followed by 3 days of intubation, along with medical management with remdesivir and a steroid course. No physical therapy was offered or employed while the patient was admitted to the hospital. The patient was subsequently discharged home. During the telemedicine visit 1 week after discharge, patient endorsed myalgia, described as ‘tightness’ and ‘spasms’, in her neck and shoulder with some radiation down to bilateral arms. Patient was encouraged to do home exercises, which she had previously learnt from physical therapy. The patient was seen in clinic 1 month later for medication refill. Patient continued to endorse intermittent ‘achy, spasms’ pain in her neck, shoulder area. Upon physical exam, patient seemed to have palpable taut muscle bands in the trapezius and levator scapulae bilaterally, which were painful on deep palpation and triggered her pain pattern. Patient underwent a series of ten trigger point injections (dry needling) in her trapezius and levator scapulae, with about a 50% immediate relief. Patient was referred to physical therapy to regain strength and conditioning targeted specially for her neck and upper back. On her post-procedure follow-up 4 weeks later, patient's numerical pain rating scale (NPRS) was a 2/10 and seemed to have had a significant improvement and reduction in her self-reported pain by 75%.

## Case patient #2

Patient is a 35-year-old female with history of congenital scoliosis s/p Harrington bar fixture following chronic pain management for chronic mid-lower back pain due to thoracic and lumbar spondylosis. Patient's pain was well controlled on naproxen 500 mg BID PRN along with regular at home physical therapy and aquatherapy. Patient was diagnosed with SARS-CoV-2 by PCR test in July 2020. She was advised to self-quarantine at home and only seek emergency care if symptoms deteriorate. Along with respiratory symptoms, patient started experiencing bilateral shoulder blade pain radiating to her shoulders 3 days after testing positive for SARS-CoV-2. Her muscle pain got worse around day 5. Patient recalled that she “just did not want to do anything” and had “been in bed” since her diagnosis. Patient was seen 1 month post SARS-CoV-2 diagnosis at which time she continued to endorse pain along her shoulder bilaterally, albeit not as severe in nature; NPRS was 6/10. On physical exam, taut palpable muscle bands were palpated in the inferior spinatus muscle, with pain referred to her anterior deltoids bilaterally. Patient wanted to pursue conservative management initially. She was provided with stretches for her neck/shoulder and advised to do them daily. Patient was subsequently followed up in clinic 3 months later with some improvement. The NPRS at this follow-up visit was 4/10. On physical exam, some palpable taut muscle bands were still identified in the inferior spinatus muscle, with similar referral pattern as before. Patient still refused interventional management in the form of trigger point injections.

## Case patient #3

Patient is a 71-year-old overweight male with history of lumbar spondylosis, lumbar radiculopathy, and failed back surgery syndrome who was being managed in the chronic pain clinic with gabapentin 600 mg TID, meloxicam 7.5 mg BID PRN, along with tizanidine 2 mg TID PRN. Patient was diagnosed with SARS-CoV-2 by a PCR test in July 2020, went to the emergency room briefly but was told to go back home to quarantine as he did not require supplemental oxygen therapy and did not require steroids or antiviral therapy for SARS-CoV-2 therapy. Patient followed up in chronic pain clinic 2 months after SARS-CoV-2 diagnosis at which time patient endorsed new neck pain, for about 1 month, “sharp pins” in his neck bilaterally, with radiation to his scalp causing headaches intermittently. NPRS was 6/10. Patient stated he had never had neck pain before and had no prior history of migraine headaches. Physical exam was significant for palpable taut bands (total of 6) in the trapezius bilaterally, with pain referred to the occipital region. Patient chose to proceed with trigger point injections (dry needling) at these taut muscle bands with immediate confirmation of the myofascial trigger point and about 40–50% improvement in pain immediately. Patient reported NPRS of 3/10 during 4-week post procedure follow-up.

## Discussion

Myofascial pain is a widespread cause of soft tissue pain, tenderness or autonomic changes. Clinically, there is an assessment of the patient's history such as age, location of pain, duration, daily activity level, occupation and other associated symptoms to narrow down the condition. Traumatic events, muscular overloads or psychological stress may lead to development of one or more palpable bands in which a latent myofascial trigger point could appear [9]. The diagnosis is largely clinical with required features including as follows: taut band within the muscle, exquisite tenderness at a point on the taut band, and reproduction of the patient's pain [2]. Other features such as local twitch response, referred pain, weakness, restricted range of motion and autonomic signs may occur but are not required. However, due to patient variability and other co-existing conditions, these symptoms may often be masked. Although there are no confirmatory lab tests, there are a few examinations that can be used to pinpoint this syndrome such as electromyography, thermography, ultrasound imaging and microdialysis.

Treatment of myofascial pain requires eliminating the initial factors that caused the trigger point to occur by stretching sarcomeres and restoring normal muscle length. In that sense, there have been hands on therapies that have been described such as utilizing muscle energy, ischemic compression, strain and counterstrain, trigger point pressure and transverse friction massage to inactivate the myofascial trigger point. All of these therapies have been noted to provide moderate relief for brief periods of time [10]. Noninvasive, nonmanual therapies such as electrical stimulation (transcutaneous electric nerve stimulation), ultrasound, laser and magnet therapies have been described with moderate evidence for short and long-term relief [11]. Invasive therapies include inactivating the trigger point with a trigger point injection, with or without local anesthetic [12]. Inserting a needle into the trigger point results in a local twitch response, often with reproduction of pain, followed by a relaxation of taut muscle band and alleviation of pain.

Medical management consists of a multi-modal analgesic plan by using NSAIDs, diclofenac, COX-2 inhibitors, tramadol, tropisetron and lidocaine patches. Although each medication has a different mechanism of action and may not target the same specific receptors, they can be used to help alleviate pain that is similar to musculoskeletal pain. Another category includes muscle relaxants and antidepressants such as Tizanidine and Benzodiazepines and Tricyclic antidepressants, respectively [13]. Tizanidine in specific is considered a first line agent whereas TCAs may be used if other treatment options fail due to the high side effect profile [14]. Botulinum type A toxin may also be used; however, data currently indicates more trials need to be done to ascertain efficacy [15].

As described above, MFPS is typically instigated by traumatic stress or mechanical insult of psychological stress. SARS-CoV-2 is likely one of these instigating stressors and the common denominator among the three cases presented in this case series. MFPS was diagnosed in these patients after a thorough review of patient's history and physical exam findings. Each patient's labs were checked to rule out any inflammatory etiology as well as assessing cervical spine MRIs to rule out possible disc, facet or radiculopathy symptoms. Furthermore, the physical exam findings on these patients revealed taut tender points that reproduced similar pain to what they were feeling with symptom onset.

There is no direct correlation between SARS-CoV-2 and MFPS. However, it is plausible that long periods of bedrest (for example with prolonged hospitalization) or prolonged inactivity may lead to muscular overloads, atrophy or psychological stress and subsequently may lead to development of one or more palpable bands in which a latent myofascial trigger point could appear. Additionally, myalgia during viral infection is most commonly mediated by IL-6, whose upregulation causes muscle and joint pain [16]. It is believed that myalgia in SARS-CoV-2 infection might reflect the generalized inflammation and cytokine response, again adding insult to injury. Furthermore, some SARS-CoV-2 cases result in prolonged pain, morbidity and the resultant ‘long COVID syndrome’. SARS-CoV-2 could induce changes in nociceptor excitability that would be expected to promote pain, induce neuropathies, and possibly worsen existing pain conditions. One hypothesis is cytokine dysregulation in COVID-19 and how this unique pattern of immune reaction may derive interactions with nociceptors that would be expected to promote pain [17,18].

The term ‘long COVID syndrome’ was recently coined and describes symptoms that may last more than 12–24 weeks and ‘persistent post-COVID syndrome’ describes symptoms lasting more than 24 weeks. These symptoms have been found to include multiple organ systems that require targeted therapy and treatment. A few examples that are associated with post-COVID are autonomic dysfunctions such as chest pain and palpitations, neurocognitive dysfunctions such as dizziness and brain fog and musculoskeletal dysfunctions such as myalgias and arthralgias [19]. Due to the variety of symptomatology that coronavirus has caused, it has been stated that there should be a temporal relationship between new symptom onset and positive SARS-CoV-2 testing, which is seen in the cases above. These cases demonstrate a correlation between MFPS and coronavirus that exists past the ‘wash out’ period of 4–5 weeks after viral diagnosis. With new research arising, it is shown that if symptoms exist past this period, there is a strong relationship between diagnosis and symptoms.

## Conclusion

There is a widespread causation of coronavirus and implications on the human body; however, a newer relationship is seen between MFPS and SARS-CoV-2 as depicted in these three cases. This virus has been shown to cause strain and tension on skeletal muscles in particular, thus leading to trigger points. The long-term side effects accompanied by periods of bed rest have shown a correlation in the nature of pain symptoms following PCR diagnosis of SARS-CoV-2. It is presumed that the cytokine storm that is caused by SARS-CoV-2 can have an effect on chronic pain symptoms [20,21]. In the future, there can be a cohort study assessing patients who have had a diagnosis of coronavirus to assess for the development of trigger points or MFPS independent of other comorbidities.
